# Adipokines NUCB2/Nesfatin-1 and Visfatin as Novel Inflammatory Factors in Chronic Obstructive Pulmonary Disease 

**DOI:** 10.1155/2014/232167

**Published:** 2014-05-06

**Authors:** Sirpa Leivo-Korpela, Lauri Lehtimäki, Mari Hämälainen, Katriina Vuolteenaho, Lea Kööbi, Ritva Järvenpää, Hannu Kankaanranta, Seppo Saarelainen, Eeva Moilanen

**Affiliations:** ^1^Department of Respiratory Medicine, Tampere University Hospital, PL 2000, 33521 Tampere, Finland; ^2^The Immunopharmacology Research Group, School of Medicine, University of Tampere and Tampere University Hospital, 33014 Tampere, Finland; ^3^Allergy Centre, Tampere University Hospital, PL 2000, 33521 Tampere, Finland; ^4^Medical Imaging Centre, Tampere University Hospital, PL 2000, 33521 Tampere, Finland; ^5^Department of Respiratory Medicine, Seinäjoki Central Hospital, 60220 Seinäjoki, Finland

## Abstract

COPD (chronic obstructive pulmonary disease) is a common lung disease characterized by airflow limitation and systemic inflammation. Recently, adipose tissue mediated inflammation has gathered increasing interest in the pathogenesis of the disease. In this study, we investigated the role of novel adipocytokines nesfatin-1 and visfatin in COPD by measuring if they are associated with the inflammatory activity, lung function, or symptoms. Plasma levels of NUCB2/nesfatin-1 and visfatin were measured together with IL-6, IL-8, TNF-**α**, and MMP-9, lung function, exhaled nitric oxide, and symptoms in 43 male patients with emphysematous COPD. The measurements were repeated in a subgroup of the patients after four weeks' treatment with inhaled fluticasone. Both visfatin and NUCB2/nesfatin-1 correlated positively with plasma levels of IL-6 (*r* = 0.341, *P* = 0.027 and rho = 0.401, *P* = 0.008, resp.) and TNF-**α** (*r* = 0.305, *P* = 0.052 and rho = 0.329, *P* = 0.033, resp.) and NUCB2/nesfatin-1 also with IL-8 (rho = 0.321, *P* = 0.036) in patients with COPD. Further, the plasma levels of visfatin correlated negatively with pulmonary diffusing capacity (*r* = −0.369, *P* = 0.016). Neither of the adipokines was affected by fluticasone treatment and they were not related to steroid-responsiveness. The present results introduce adipocytokines NUCB2/nesfatin-1 and visfatin as novel factors associated with systemic inflammation in COPD and suggest that visfatin may mediate impaired pulmonary diffusing capacity.

## 1. Introduction

Chronic obstructive pulmonary disease (COPD) is a disorder characterized by persistent airflow limitation with systemic manifestations [1]. In addition to the inflammatory process in the lungs, there is a low-grade systemic inflammation which is linked to the pathogenesis of the comorbidities present in COPD [2–4]. It is not known whether systemic inflammation is a spillover of the inflammation present in the lungs or if pulmonary manifestations are one form of expression of this systemic disease [3, 5]. Smoking, lung hyperinflation, tissue hypoxia, and skeletal muscle dysfunction have been suggested as possible factors involved in the pathogenesis of the systemic inflammation in COPD [2]. Recently, adipose tissue mediated inflammation has gathered increasing interest as a significant mechanism in inducing and promoting systemic inflammation in COPD [4].

Adipokines (also known as adipocytokines) are protein mediators secreted by adipose tissue and they are involved not only in the regulation of energy metabolism but also in inflammatory responses in many chronic inflammatory diseases [6, 7]. There is increasing body of evidence supporting a significant role of adipokines adiponectin and leptin in the inflammatory processes in COPD [8], but only a little or nothing is known about the other adipokines like nesfatin-1 and visfatin in inflammatory lung diseases. So far, there are no previous publications on nesfatin-1 and only a few reports on visfatin in COPD.

Nesfatin-1 is a novel adipokine discovered in 2006 and at first linked to appetite and body weight control in rats [9]. Nesfatin-1 is expressed in human adipose tissue and TNF-*α*, IL-6, insulin, and dexamethasone have been shown to increase its secretion [10]. Nesfatin-1 has also been shown to regulate inflammatory responses and cell apoptosis in rats [11] and to have cardioprotective effects in human studies [12]. There are only two previous publications on nesfatin-1 in lung diseases: one concerning changes in fat mass in lung cancer [13] and the other on cystic fibrosis [14].

Visfatin, also known as nicotinamide phosphoribosyltransferase (NAMPT) and previously identified as pre-B cell colony-enhancing factor (PBEF), was originally discovered in lymphocytes, bone marrow, liver, and muscle [15]. Later, visfatin was identified also in the lungs [16], and, interestingly, the proinflammatory cytokine IL-1 has been reported to enhance visfatin expression in pulmonary epithelial and endothelial cells* in vitro* [17]. Also proinflammatory cytokines IL-6 [18] and TNF-*α* [19] have been shown to induce the expression of visfatin. Granulocytes and monocytes are major sources of visfatin [20], and it is also produced by macrophages and adipocytes [21]. Visfatin is a proinflammatory cytokine involved in the regulation of inflammation and innate immunity [22, 23]. In the lungs, visfatin is associated with acute lung injury (ALI) [24] and the inhibition of visfatin synthesis has been shown to attenuate inflammation and apoptosis associated with severe virus infection in lung endothelium [25].

In the present study, we measured the plasma levels of visfatin and NUCB2/nesfatin-1 in patients with COPD and in controls and investigated if these adipokines are associated with other markers of inflammation, lung function, the degree of emphysema, and symptoms or with the response to inhaled glucocorticoids in patients with COPD.

## 2. Materials and Methods

### 2.1. Subjects and Study Design

Forty-three steroid-naïve male patients with COPD were recruited among subjects referred from primary care for diagnostic assessment to the Department of Respiratory Medicine at Tampere University Hospital, Tampere, Finland. COPD diagnosis was based on GOLD strategy paper [1] and the inclusion criteria were smoking history of at least 20 pack-years, symptoms of COPD (cough, sputum production, and dyspnoea), postbronchodilator FEV_1_/FVC < 0.7, reversibility of FVC or FEV_1_ induced by ß_2_-agonist < 12% or 200 mL, and pulmonary emphysema visible on high resolution computed tomography (HRCT) of the lungs. Patients with a diagnosis or clinical history of asthma or diabetes were excluded. Ten (23%) of the patients had hypertension and five (12%) had hypercholesterolemia while the number of patients with other diseases was too small for any statistical analysis. Forty-one age-matched nonsmoking healthy males with normal lung function served as controls.

Spirometry, fractional exhaled nitric oxide concentration (FENO), and pulmonary diffusing capacity per unit of alveolar volume standardized for haemoglobin concentration (Hb-*D*_L,CO_/*V*_*A*_) were measured, high resolution computed tomography (HRCT) of the lungs was performed, and symptoms were scored with St. George's Respiratory Questionnaire (SGRQ) in patients with COPD. A venous blood sample was drawn in both groups. The same measurements excluding HRCT were repeated in twenty-seven patients with COPD after 4 weeks of treatment with inhaled fluticasone propionate (Flixotide Diskus 500 *μ*g b.i.d.; GlaxoSmithKline, Ware, UK).

### 2.2. Adipokines and Inflammatory Markers

Plasma concentrations of NUCB2/nesfatin-1, visfatin, interleukin 6 (IL-6), interleukin 8 (IL-8), tumor necrosis factor alpha (TNF-*α*), and matrix metalloproteinase 9 (MMP-9) were determined by enzyme immunoassay by using the following reagents: NUCB2/nesfatin, TNF-*α*, and MMP-9: R&D Systems Europe Ltd., Abingdon, UK; visfatin: Phoenix Pharmaceuticals Inc., Karlsruhe, Germany; IL-6: Sanquin, Amsterdam, Netherlands; and IL-8: BD Biosciences, San Diego, CA, USA. According to the manufacturer, the nesfatin-1 antibody detects the protein nucleobinding-2 (NUCB2) in addition to nesfatin-1 which is derived from NUCB2 by posttranslational processing. Therefore, the term NUCB2/nesfatin-1 is used when referring to our own measurements. The detection limits and interassay coefficients of variation were 7.8 pg/mL and 11.6% for NUCB2/nesfatin-1, 0.1 ng/mL and 8.3% for visfatin, 0.3 pg/mL and 7.6% for IL-6, 1.56 pg/mL and 7.0% for IL-8, 0.5 pg/mL and 6.5% for TNF-*α*, and 7.8 pg/mL and 6.0% for MMP-9.

### 2.3. Lung Function, Exhaled NO, HRCT, and Symptom Scoring

Spirometry was measured (Vmax 20C, Sensor-Medics, Yorba Linda, CA, USA) before and after 400 *μ*g of inhaled salbutamol. Pulmonary diffusing capacity was measured with Vmax 20C, Sensor-Medics.

Fractional exhaled nitric oxide concentration at exhalation flow rate of 50 mL/s (FENO_0.05_) was measured with a Sievers NOA 280 NO-analyzer (Sievers Instruments, Boulder, CO, USA) as previously described [26].

Airway wall thickness and the extent of emphysema on pulmonary high resolution computed tomography (HRCT) (Siemens Somatom Plus 4, Siemens Medical, Erlangen, Germany) were assessed by two experienced thoracic radiologists (Ritva Järvenpää and Lea Kööbi) as previously described [27].

The subjects filled in the Finnish translation of St. George's Respiratory Questionnaire (SGRQ) containing questions and scoring on three aspects of the disease (symptom frequency and severity, activities that cause or are limited by breathlessness, and the impact of the disease on social functioning including psychological disturbances resulting from the disease) to obtain a total score. The scale has a range from 0 to 100, with higher scores representing more severe disease.

### 2.4. Statistics

Visfatin was normally distributed, while the distribution of NUCB2/nesfatin-1 was skewed and could not be normalised after log-transformation. *t*-test or Mann-Whitney test was used to examine differences between healthy controls and patients with COPD, where appropriate. Pearson's *r* or Spearman's rho was used to analyse the correlations between adipokines and other inflammatory markers or markers of disease severity, where appropriate. Changes in plasma levels of adipokines and other measures during fluticasone treatment were analysed with paired *t*-test or Wilcoxon's test, where appropriate. The results are presented as mean ± SD for normally distributed data and as median (interquartile range) for nonnormally distributed data. SPSS 19 software (SPSS Inc., Chicago, Illinois, USA) was used in the statistical analysis.

### 2.5. Ethics

The study was approved by the Ethics Committee of Tampere University Hospital, Tampere, Finland, and complies with the declaration of Helsinki. All subjects provided their written informed consent.

## 3. Results

### 3.1. Characteristics of the Subjects

In the patients with COPD, the mean age was 59.5 ± 7.8 (mean ± SD) years and the mean forced expiratory volume in 1 second (FEV_1_) was 53 ± 14% of the predicted. The age- and sex-matched healthy controls had normal lung function and there were no significant differences in BMI between the patients with COPD and the controls (25.8 ± 4.2 versus 26.7 ± 3.9 kg/m^2^, resp., *P* = 0.332). Plasma levels of adipokines NUCB2/nesfatin-1 and visfatin in the patients with COPD and controls are given in Table 1. Visfatin levels were lower in COPD, but plasma NUCB2/nesfatin-1 levels did not differ from controls. Neither of the adipokines measured differed between ex-smokers (*n* = 10) and current smokers (*n* = 33) or between the patients with or without hypertension (*n* = 10) or between the patients with or without hypercholesterolemia (*n* = 5) in the COPD group.

### 3.2. Correlations between Adipokines and Other Parameters

Correlations between adipokines and other parameters in patients with COPD are given in Table 2. Both NUCB2/nesfatin-1 and visfatin correlated with IL-6 in the patients with COPD but not in controls. Therefore, two other proinflammatory cytokines, that is, TNF-*α* and IL-8, were also measured in patients with COPD. Interestingly, NUCB2/nesfatin-1 and visfatin correlated positively with TNF-*α* and NUCB2/nesfatin-1 also with IL-8. A negative correlation was seen between visfatin and pulmonary diffusing capacity (Hb-*D*_L,CO_/*V*_*A*_) suggesting that visfatin is associated with parenchymal impairment. The adipokines did not correlate with BMI, radiological changes, levels of MMP-9, exhaled nitric oxide, or symptoms score.

### 3.3. The Treatment Responses

Four weeks of treatment with inhaled fluticasone caused no significant changes in plasma levels of NUCB2/nesfatin-1 (before: 75.0 (18.3–119.4) pg/mL, after: 61.1 (17.5–116.1) pg/mL, *P* = 0.399) or visfatin (before: 7.9 ± 1.5 ng/mL, after: 8.0 ± 1.4 ng/mL, *P* = 0.804). As expected, the treatment decreased St. George's Respiratory Questionnaire total score describing the impact of the disease (before: 36.4 ± 15.6, after: 30.8 ± 16.0, *P* = 0.015) and symptom score (before: 55.2 ± 22.1, after: 38.0 ± 23.2, *P* < 0.001). The baseline plasma levels of NUCB2/nesfatin-1 or visfatin did not correlate with the degree of fluticasone induced changes in either symptoms or lung function in COPD (data not shown).

## 4. Discussion

The main findings in the present study were that NUCB2/nesfatin-1 and visfatin correlated positively with systemic inflammation and, further, visfatin was associated with parenchymal impairment in patients with emphysematous COPD. This suggests that NUCB2/nesfatin-1 and visfatin may have a role in the inflammatory processes in COPD.

Originally, adipose-tissue-derived adipokines were found to regulate energy metabolism and to be associated with the chronic low-grade inflammation present in obesity-related metabolic disturbances and inflammatory diseases [28, 29]. Later adipokines have also been linked to inflammatory lung diseases like asthma and COPD [8] and the majority of the studies have concentrated on the role of leptin and adiponectin in these diseases. It has been suggested that high circulating leptin and low adiponectin predict asthma independent of obesity and that low leptin and high adiponectin are associated with stable COPD [30]. The results on the association between adipokines and COPD are, however, still conflicting and the studies are not covering all adipokines such as visfatin, which is known to be associated with other chronic inflammatory and destructive syndromes such as rheumatic diseases [31].

We found that NUCB2/nesfatin-1 and visfatin concentrations in plasma correlated with circulating levels of IL-6 and TNF-*α* and NUCB2/nesfatin-1 also with IL-8 suggesting that these adipokines may have a role in the systemic inflammation in COPD. Neutrophils and macrophages are significant cell types in the pathophysiology of COPD [32]. Macrophages are also major sources of IL-6 and TNF-*α* [32] and neutrophils of IL-8 [33]. As activated macrophages also secrete adipokines [6], the association between adipocytokines nesfatin-1 and visfatin and proinflammatory cytokines IL-6 and TNF-*α* in COPD may be linked to the activation of macrophages. Further, IL-6 has been shown to induce the expression of both visfatin [18] and nesfatin-1 [10], and additionally it has been reported that TNF-*α* is able to provoke both nesfatin-1 [10] and visfatin secretions [19]. Moreover, it has been presented that visfatin itself can induce the production of TNF-*α* and especially IL-6 [22]. Also, previous reports have shown that visfatin correlates positively with IL-6 [34] and with CRP and TNF-*α* without association with BMI in patients with COPD [35], but the current study is the first report on NUCB2/nesfatin-1 in COPD.

Interestingly, we also found a negative correlation between visfatin and pulmonary diffusing capacity suggesting that visfatin is associated with parenchymal impairment in emphysematous COPD. In COPD, diffusing capacity may be decreased due to loss of alveolar surface and due to impaired diffusivity because of inflammation and oedema in the alveolar walls. As visfatin levels were not associated with the degree of emphysema visible on HRCT, we suggest that visfatin is related to the inflammatory activity impairing pulmonary diffusing capacity. This is supported by the previous findings that inflamed endothelium as well as lung epithelial cells can produce visfatin [17, 36] which may promote and amplify lung inflammation and parenchymal vascular damage present in emphysema [37].

Compared to controls, both higher [35] and lower [38] plasma visfatin levels have been reported in COPD. In the current study, plasma visfatin levels were lower in the slightly overweight men with COPD compared to healthy controls with similar BMI. Consistent with our result, significantly lower visfatin levels in normal weight or slightly overweight men with COPD [38] have been reported, while underweight men with COPD had increased visfatin levels [35]. Visfatin is expressed in visceral adipose tissue and higher [20], lower [39], and unchanged [40] circulating visfatin levels have been reported in obese compared to normal weight persons. Accordingly, we and others [35] have not found any correlation between visfatin and BMI in patients with COPD. The higher visfatin in underweight COPD patients reported by Liu and coworkers [35] might be explained by the fact that COPD patients with lower BMI have usually more severe systemic inflammation [41] and that visfatin itself can inhibit neutrophil apoptosis [42] and thus enhance lung inflammation in COPD patients. Liu et al. also hypothesized that hypoxemia present in severe COPD may contribute to increased visfatin [35].

Neither visfatin nor NUCB2/nesfatin-1 concentrations were changed during inhaled fluticasone treatment in the current study. This may be explained by the fact that a short term treatment with inhaled fluticasone likely has no significant systemic anti-inflammatory effect, and this is also supported by the present finding that neither IL-6 nor MMP-9 levels changed during the treatment. As far as we know, there are no other studies on the effect of inhaled glucocorticoids on the levels of NUCB2/nesfatin-1 and visfatin, but in previous studies systemic glucocorticoid treatment did not alter circulating visfatin levels in humans [43, 44]. Further, in the current study neither of these adipokines was associated with the treatment responses assessed by lung function or symptoms. However, in a previous study we found that high levels of adiponectin were associated with steroid-responsiveness in COPD [27].

## 5. Conclusions

The present results introduce adipocytokines NUCB2/nesfatin-1 and visfatin as novel inflammatory factors in stable emphysematous COPD. Furthermore, visfatin was also associated with reduced pulmonary diffusing capacity. The findings suggest that NUCB2/nesfatin-1 and visfatin have a proinflammatory role in the pathogenesis of emphysematous COPD, but further studies are needed to evaluate if adipokines could be used as biomarkers for phenotyping or subgrouping patients with COPD or as anti-inflammatory drug targets.

## Figures and Tables

**Table 1:** Plasma levels of adipokines and other inflammatory markers in patients with COPD and healthy controls.

	COPD	Controls	*P* value
*N*	43	41	
NUCB2/nesfatin-1 (pg/mL)	75.0 [26.2–103.1]	43.1 [17.9–86.6]	0.117
Visfatin (ng/mL)	7.5 ± 1.5	8.9 ± 2.3	**0.002**
IL-6 (pg/mL)	1.5 ± 1.3	1.5 ± 1.2	0.888
MMP-9 (ng/mL)	40.6 ± 17.3	33.9 ± 12.6	**0.048**

Values are presented as mean ± SD for normally distributed data and as median [interquartile range] for nonnormally distributed data.

IL-6: interleukin 6; MMP-9: matrix metalloproteinase 9.

**Table 2:** Correlations between adipokines and other parameters in patients with COPD (*n* = 43).

	NUCB2/nesfatin-1	Visfatin
FEV_1_ (% pred)	rho = 0.097	*r* = 0.127
	*P* = 0.535	*P* = 0.422

FENO_0.05_ (ppb)	rho = −0.035	*r* = 0.040
	*P* = 0.823	*P* = 0.802

Hb-*D*_L,CO_/*V*_*A*_ (% pred)	rho = −0.103	*r* = −0.369
	*P* = 0.512	*P* = 0.016

Emphysema percentage (%)	rho = 0.076	*r* = 0.204
	*P* = 0.626	*P* = 0.194

IL-6 (pg/mL)	rho =** 0.401**	*r* = 0.341
	*P* = 0.008	*P* = 0.027

IL-8 (pg/mL)	rho = **0.321**	*r* = 0.121
	*P* = 0.036	*P* = 0.443

TNF-*α* (pg/mL)	rho =** 0.329**	*r* = 0.305
	*P* = 0.033	*P* = 0.052

The distribution of nesfatin-1 was skewed while visfatin was normally distributed; therefore Spearman's rho and Pearson's *r* were used to analyze the correlations of NUCB2/nesfatin-1 and visfatin, respectively, to the other parameters presented.

FEV_1_: forced expiratory volume in 1 second; FENO_0.05_: fractional exhaled nitric oxide concentration at exhalation flow rate of 50 mL/s; Hb-*D*_L,CO_/*V*_*A*_: diffusing capacity for carbon monoxide per unit of alveolar volume standardized for haemoglobin; IL: interleukin; TNF: tumor necrosis factor.
